# Topological states on the gold surface

**DOI:** 10.1038/ncomms10167

**Published:** 2015-12-14

**Authors:** Binghai Yan, Benjamin Stadtmüller, Norman Haag, Sebastian Jakobs, Johannes Seidel, Dominik Jungkenn, Stefan Mathias, Mirko Cinchetti, Martin Aeschlimann, Claudia Felser

**Affiliations:** 1 Max Planck Institute for Chemical Physics of Solids, 01187 Dresden, Germany; 2 Max Planck Institute for the Physics of Complex Systems, 01187 Dresden, Germany; 3 School of Physical Science and Technology, ShanghaiTech University, Shanghai 200031, China; 4 Department of Physics and Research Center OPTIMAS, University of Kaiserslautern, 67653 Kaiserslautern, Germany; 5 I. Physikalisches Institut, Georg-August-Universität Göttingen, 37077 Göttingen, Germany

## Abstract

Gold surfaces host special electronic states that have been understood as a prototype of Shockley surface states. These surface states are commonly employed to benchmark the capability of angle-resolved photoemission spectroscopy (ARPES) and scanning tunnelling spectroscopy. Here we show that these Shockley surface states can be reinterpreted as topologically derived surface states (TDSSs) of a topological insulator (TI), a recently discovered quantum state. Based on band structure calculations, the *Z*_2_-type invariants of gold can be well-defined to characterize a TI. Further, our ARPES measurement validates TDSSs by detecting the dispersion of unoccupied surface states. The same TDSSs are also recognized on surfaces of other well-known noble metals (for example, silver, copper, platinum and palladium), which shines a new light on these long-known surface states.

## Abstract (web summary)

The surfaces of noble metals possess Shockley states which exhibit Rashba-type spin splitting and spin-momentum locking. Here, the authors use *ab initio* methods and photoemission spectroscopy to demonstrate how such Shockley states may be reinterpreted as topologically protected surface states.

## Graphical abstract

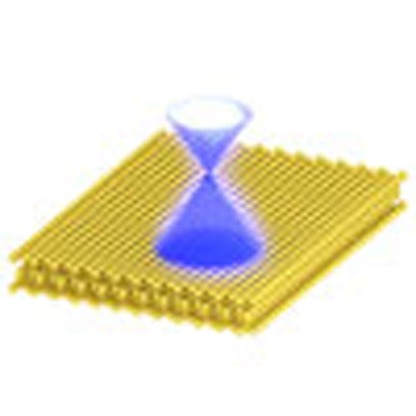

The history of surface states (SSs)^1^,^2^,^3^,^4^,^5^,^6^,^7^,^8^,^9^,^10^ can be traced back to 1932 when Tamm^11^ predicted the existence of special electronic states near the crystal boundary. Soon Shockley^3^ found that SSs, usually called Shockley SSs later, emerge in an inverted energy gap owing to band crossing, for which the symmetry of the bulk band structure was found to be crucial^12^. The SSs on (111)-oriented surfaces of noble metals (for example, Au, Ag and Cu) have been known as typical Shockley-type SSs (for example, refs 13, 14, 15), wherein SSs appear inside an inverted energy gap of *s* and *p* bands at the centre of the surface Brillouin zone (BZ). As a result of inversion symmetry breaking on the surface, these SSs exhibit Rashba-type^16^ spin splitting with spin-momentum locking at the Fermi surface^2^,^15^,^17^,^18^, which is essential for spintronic devices^19^. These SSs have been used to design quantum corrals^20^,^21^,^22^,^23^,^24^ as well as artificial Dirac fermions^25^ and benchmark the capability of angle-resolved photoemission spectroscopy (ARPES)^4^,^5^ and scanning tunnelling spectroscopy (STS)^6^. As another intrinsic SS, the topological SS (TSS) has recently attracted great research interest in the condensed matter physics community^9^,^10^. Metallic TSSs inside the bulk energy gap are induced by the topology of the inherent bulk band structure, which can be understood as an inversion between the conduction and valence bands that have opposite parities^26^,^27^. TSSs have been predicted and observed in many compounds^28^, such as HgTe (refs 26, 29) and Bi_2_Se_3_ (refs 30, 31), wherein spin and momentum are locked up and form spin texture in the Dirac-cone-like band structure. From a naive viewpoint of the band inversion, Shockley SSs and TSSs are not fully exclusive of each other. TSSs of Bi_2_Se_3_, for example, have been described in a generalized Shockley model that includes spin-orbit coupling (SOC) by Pershoguba *et al*.^32^ This inspires us to pose the opposite question: Can some Shockley SSs be understood as TSSs?

In this work, we revisit SSs on Au, Ag and Cu (111) surfaces by *ab initio* band structure calculations and ARPES performed on the Au(111) surface with a momentum microscope^33^ for the detection of the complete angular distribution of the photoemitted electrons as function of their kinetic energy. We find that these famous Shockley SSs are also TSSs, which originate from the inverted bulk band structure. The Rashba-split-like energy dispersion can be regarded as a strongly distorted Dirac cone. Although noble metals do not exhibit an energy gap, a *Z*_2_ topological invariant *ν*_0_=1 can be well defined owing to the existence of a direct energy gap above the Fermi energy. Thus, the existence of TSSs is generalized from the insulators to common metals. Besides providing a new understanding of noble metal SSs, finding topological states on late transition metals will provoke interesting questions on the role of topological effects in surface-related processes, such as adsorption and catalysis.

## Results

### Comparison of different types of SSs

**Figure 1:**
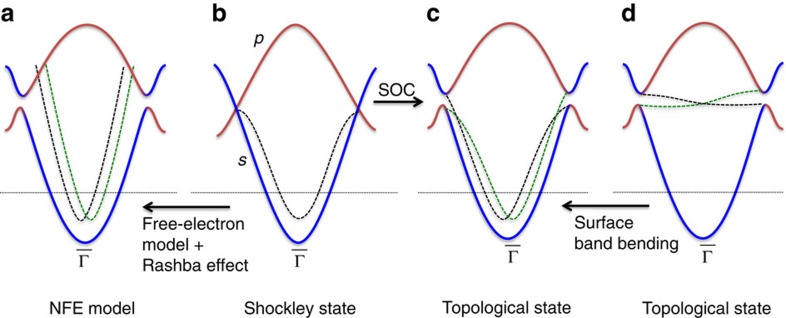
Relations between Shockley states and topological surface states. (**a**) The commonly employed nearly-free-electron (NFE) model to describe Shockley states where Rashba-type spin-split exists. It applies for the occupied states only near the zone centre, but neglects the topology of band structure that requires the whole momentum space. Blue and red solid lines stand for bulk valence (*s* state) and conduction (*p* state) bands, respectively. Black and green dashed lines stand for surface states with opposite spin polarization. The Fermi energy (horizontal dashed line) crosses the middle of bulk valence bands and the bottom of surface states. (**b**) The original Shockley model without considering SOC. In the inversion gap between conduction and valence bands, spin-degenerate Shockley surface states emerge. (**c**) Topological surface states evolved from Shockley states. Due to SOC, the bulk gap opens and the original Shockley state split into two branches of topological surface states, each of which disperses from the bulk valence to conduction bands. (**d**) The topological surface states commonly exist inside the inverted bulk gap with a simple Dirac-cone-type dispersion. For gold, strong surface band bending heavily deforms the Dirac cone by pulling the Dirac point below the Fermi energy.

Shockley states on gold surface have been commonly modelled as a nearly-free-electron (NFE) model^34^. They exhibit quadratic energy dispersion with the spin-split that is explained by the Rashba effect, as illustrated in Fig. 1. The NFE model fits well to the band structure near the surface BZ centre 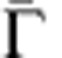, which are the occupied states detected by ARPES. However, this model neglects information of the whole BZ, that is, the topologcial properties of the band structure. Actually, recent inverse photoemission measurement^35^ on the unoccupied states already indicated the discrepancy between NFE model and SS dispersions in the region far away from 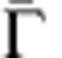. We should note that the original Shockley's theory^3^ implies some information of topology. It predicts SSs inside the inverted energy gap without considering SOC for gold (Fig. 1b), in which the bulk is gapless at the *s*–*p* band-crossing point and SSs are spin degenerate. By introducing SOC, we find two simple consequences in band structure: The bulk opens a direct energy gap at the band-crossing point and SSs become clearly TSSs. Although they can still be fitted by a NFE model only near 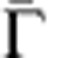, TSSs disperse from the valence to conduction bands inside the direct gap across the whole BZ, which is verified by our following ARPES measurement on unoccupied states. Compared with TSSs commonly observed with a simple Dirac-cone-like dispersion (see Fig. 1d), the surface band bending pulls the Dirac point below the Fermi energy, strongly deforming the shape of the Dirac cone. However, this can not remove the topological nature of gold SSs. We note that one signature of the Dirac cone deformation is the crossing of two spin channels of TSSs in high-energy region away from the 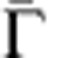 point (see Fig. 1c), as revealed by our following calculations.

### Bulk band structure

**Figure 2:**
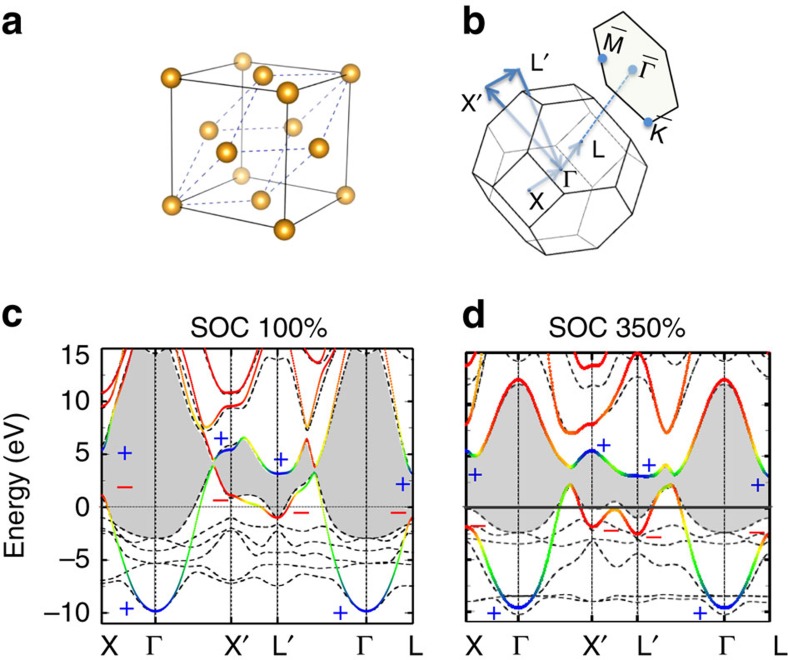
Crystal structure and band structures of bulk gold. (**a**) Face-centred cubic lattice structure. The dashed lines indicate the primitive unit cell that includes only one atom (the yellow sphere). (**b**) The first Brillouin zone (BZ) of the bulk and the surface BZ projected onto the (111) surface. The band structure of the bulk with (**c**) normal SOC strength (100%) and (**d**) enhanced SOC to 350%. Band structures are plotted along the high symmetry lines *X*(0.5 0.5 0)−Γ (0 0 0)−*X*′(0.5−0.5 0)−Γ−*L*′(0.5−0.5 0.5)−Γ−*L* (0.5 0.5 0.5), in which all coordinates are given in units of reciprocal lattice vectors. Dashed black lines are *ab initio* results while colour lines are projection to Au-*sp* states through Wannier functions. The colour gradient from blue to red represents varying contributions from *s* to *p* states. The parity eigen values of *s*-type (blue, ‘+') and *p*-type (red, ‘−') wave functions are labelled in the band structure. The grey shadow region indicates the topological energy gap that are induced by the *sp* band inversion. The Fermi energy is shifted to zero and indicated by the horizontal dotted line. The *s* and *p* bands get inverted in order at *X*(*X*′) and *L* (*L*′) points while they remain in the normal order only at the Γ point, which makes the band structure topologically nontrivial with *Z*_2_ index *ν*_0_=1.

Noble metals share the same face-centred-cubic (FCC) lattice structure. We take Au as an example. As shown in Fig. 2a, the primitive unit cell includes a single Au atom as the inversion centre of the lattice. Therefore, the parity of the Bloch wave function is consistent with that of the corresponding atomic orbital: ‘+' for the Au-*s* and *d* orbitals and ‘−' for the Au-*p* orbital. The bulk band structure that includes the SOC effect is shown in Fig. 2c. The Fermi energy crosses the middle of a band wherein *s* (blue colour) and *p* (red colour) states hybridize together, wherein the *d* bands are fully occupied and below the Fermi energy. Because the band inversion only involves *sp* rather than *d* states, we project the band structure to Au-*sp* states using Wannier function method for simplicity. Above the half-filled band, a direct energy gap exists in the whole BZ owing to SOC, as indicated by the grey shadow in Fig. 2c, though the indirect gap is still zero. As we will see, this direct energy gap determines the topology of SSs. For the sake of simplicity, we call bands below and above the gap valence and conduction bands, respectively. To illustrate the band inversion clearly, we show the energy dispersion along Γ−*X*′−*L*′−Γ lines, wherein *X*′(*L*′) is equivalent to *X*(*L*). Because of relativistic contraction of Au-6*s* orbitals (ref. 36, and references therein), the *s* band is lower in energy than the *d* and *p* bands at the Γ point. In contrast, the *s* band energy is even higher than that of *p* bands at *X*′(*X*) and *L*′(*L*) points. Thus, one can find that *s* and *p* bands preserve the normal order at the Γ point but get inverted at other time-reversal invariant momenta (TRIM) *X*′(*X*) and *L*′(*L*) points. Between the Γ and *X*′(*L*′) points, the *s* and *p* bands cross each other. The direct energy gap opens at the crossing point because of band anticrossing caused by SOC. The *Z*_2_ topological invariant *ν*_0_ of a topological insulator (TI) can be calculated by the product of the parity eigenvalues of all valence bands at all TRIM^27^. If the parity product is 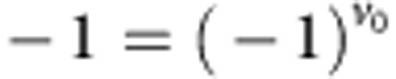, then *ν*_0_=1 represents a TI; otherwise, *ν*_0_=0 represents a trivial insulator. In the FCC BZ, eight TRIM include one Γ point, three *X* points and four *L* points. Because *s* and *d* states are always ‘+' in parity while only *p* states are ‘−', the parity product at a given *k* point is determined by the number of *p* states. Therefore, the parity product of the Γ point is ‘+' since only *sd* states appear in the valence bands. However, the parity products at *X* and *L* are ‘−', for there is one *p* state as the top valence band for both *X* and *L* points. Thus, the total parity product for eight TRIM is ‘−', that is, the *Z*_2_ invariant *ν*_0_=1, showing the topologically nontrivial feature. One can see that the topology of the band structure is caused by the *s*–*p* band inversion above the Fermi energy, which is related to the relativistic contraction of the Au-6*s* state.

### Topological SSs

**Figure 3:**
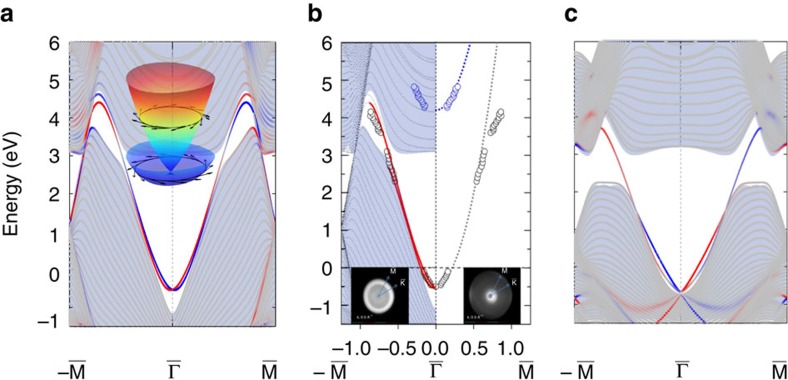
Calculated and experimental surface states. (**a**) The surface band structure of the Au(111) surface calculated for a normal SOC strength (100%). Blue and red lines indicate the topological surface states with opposite spin-polarizations. The inset illustrates the three-dimensional plot near the Dirac point with the helical spin texture. The bulk projections are indicated by the light blue shadow. (**b**) ARPES results. The dispersion of the Au(111) surface state (empty black circles) is superimposed to the theoretical prediction for normal SOC (blue and red curves). The experimental data points have been extracted from the 2PPE ARPES measurements performed with the OPO laser system combined with the momentum microscope. This experimental set-up allows recording maps with the angular distribution of the photoelectrons at constant kinetic energy (*E*_kin_) values for all available kinetic energies. The two maps corresponding to *E*_kin_=2.6 and *E*_kin_=4.0 eV are shown exemplary in the inset of **b**. The blue data points illustrate the dispersion of the image potential resonance. The spherical shape of the SS and the image potential state in momentum space lead to identical dispersions for all momentum space directions. Therefore, identical data points of the dispersion relation are shown for both high symmetry directions in **b**. (**c**) The surface band structure calculated for an enhanced SOC strength of 350%. The Fermi energy is shifted to zero.

The nontrivial *Z*_2_ topological number guarantees the existence of TSSs on the boundary. On the Au(111) surface, the *s*–*p* inversion gap (also called *L*-gap in the literature) remains at the 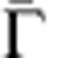 point of the surface BZ while it is reduced to zero at 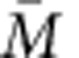 and 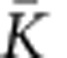 points. Near the 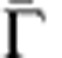 point, a pair of TSSs exist inside the *s*–*p* inversion gap with the Dirac point lying below the Fermi energy, as shown in Fig. 3a. Although the topological energy gap is above the Fermi energy, the local surface potential pulls the Dirac cone below the Fermi energy (see Supplementary Figs 1 and 2). As shown in Fig. 3a, the Dirac cone is strongly distorted, with the left-hand spin texture in the upper cone being similar to known TIs^37^,^38^. Due to the Dirac cone deformation, two spin channels of TSSs cross each other between the 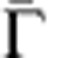 and 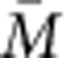 points at energy ∼3 eV above the Fermi energy. We note that the spin-channel crossing is consistent with previous calculations in ref. 35. Analysis of orbital components reveals that TSSs are mainly composed by *sp* orbitals. The same energy dispersion has been previous observed using ARPES and revealed in *ab initio* calculations^2^,^17^,^15^,^39^ this dispersion was interpreted as a Rashba-type split of *sp*-derived Shockley SSs. However, in a local region (for example, near the 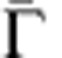 point) in the BZ, one cannot distinguish TSSs from trivial Rashba SSs. These Rashba states are equivalent to TSSs if the Rashba-split bands are regarded as a strongly distorted Dirac cone wherein the lower cone was pushed above the Dirac point. A similar dispersion of TSSs as a dramatically deformed Dirac cone was recently observed on the surface of HgTe (ref. 40).

### Two-photon-photoemission ARPES

To prove the topological origin of these SSs, we follow a twofold strategy. First of all, we use two-photon-photoemission (2PPE) ARPES to measure the energy dispersion of the SSs of the Au(111) surface below and above the Fermi energy. By combining an optical parametric oscillator (OPO) laser system with a modern momentum microscope^33^, we are able to map the dispersion of the SSs far away from the 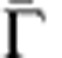 point, and confirm experimentally the strong deviation from the dispersion of a trivial Rashba SSs. Second, we demonstrate theoretically that these SSs are adiabatically connected to TSSs of a real TI. We first describe the ARPES results. To detect the electronic structure of the Au(111) surface both below and above the Fermi energy (*E*_F_), we have performed 2PPE ARPES with an OPO laser system and a momentum microscope^33^. This photoelectron analyser detects the complete angular distribution of the photoelectrons for a selected kinetic energy (*E*_kin_), as exemplarily shown in the insets of Fig. 3b for two selected values of *E*_kin_. Varying *E*_kin_ allows to record a three-dimensional data set of the ARPES intensity as function of electron momentum parallel to the surface. In this way, energy distribution curves for all high symmetry directions are recorded simultaneously (see Methods for further informations). The OPO laser system is used as excitation source for 2PPE. By varying the photon energy (between 4.13 and 4.43 eV) and the light polarization (between *s* and *p*), we can easily assign the features in the ARPES spectra to either occupied or unoccupied electronic states (that is, states below or above *E*_F_) and determine their surface- or bulk-related character. Crucially, using photon energies above 4 eV gives us access to the still unexplored region of the Brilloiun zone far away from the 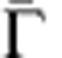 point, where we expect a strong deviation of the dispersion of the Au(111) SSs from trivial Rashba SSs. By a careful analysis of the 2PPE ARPES data (see Supplementary Figs 3 and 4) we can identify three dominant contributions to the spectra: the occupied and unoccupied part of the SSs, an unoccupied image potential resonance and bulk states. The dispersion of the SSs and of the image potential resonance are plotted in Fig. 3b with black and blue circles, respectively. Due to the perfectly spherical shape of both states in momentum space, their dispersions and hence the corresponding data points shown in Fig. 3b are identical for both high symmetry directions. From previous ARPES^15^ and STS^41^ studies it is well-known that the occupied Shockley SSs can be described by a quasi-free electron parabola with an effective mass *m*_eff_ in the range of 0.25*m*_e_ (ref. 15) to 0.37*m*_e_ (ref. 42). The significantly smaller effective mass compared with the free electron mass (*m*_e_) is due to an intrinsic coupling of the SSs with bulk states^43^. Our analysis reveals an effective mass of 0.28*m*_e_ (grey dashed line in Fig. 3b), in good agreement with previous studies. The unoccupied image potential resonance also follows a free-electron-like behaviour as it is well-known from literature^42^. The unoccupied part of the SSs, on the other hand, shows a clear deviation from the trivial free-electron-like behaviour. In particular, in the energy range from 2.0 to 3.0 eV above *E*_F_, the unoccupied SSs are found at larger *k*_||_-values than expected for a free-electron-like behaviour with *m*_eff_=0.30*m*_e_. A similar deviation in this intermediate state range was already reported previously for the Shockley SS of Au(111)^35^ and Cu(111)^43^. It was explained as the result of hybridization of the SS with the bulk bands of the noble metal. The strength of the hybridization, that is, the deviation of the experimental dispersion from the free-electron-like behaviour, increases as the SSs approach the L-band edge. Crucially, for intermediate state energies >3.0 eV the dispersion of the SSs in our data changes again and the SSs disperse faster to larger momentum values with increasing energy above *E*_F_. To our knowledge, such a strong deviation from the free-electron-like behaviour was not observed yet for SSs on fcc(111) noble metal surfaces. This observation can be explained by assuming that the SSs disperse into the bulk bands for intermediate state energies >3.0 eV to connect the valence and conduction bands. This behaviour, together with the dispersion of the SSs, is in full agreement with the calculations in Fig. 3a, pointing to the topological nature of the SSs. More details can be found in Supplementary Note 1.

### Adiabatic evolution into a TI

To conclusively demonstrate the topological nature of the SSs we now turn to the *ab initio* calculations. Here we increase the strength of SOC artificially and realize an indirect energy gap in bulk Au, for example, when the SOC strength is 350% of the normal one. The motivation for this approach is that topological nature of the SSs has been long neglected plausibly due to the lack of an energy gap in gold. Thus, we design here a real gap to demonstrate these SSs derived from topology. In the bulk, the band structures with 100% SOC strength and 350% SOC strength are adiabatically connected to each other, exhibiting the same topology with *ν*_0_=1. On the surface, an energy gap opens in the band structure for the 350% SOC case (Fig. 2d). Inside this gap, a pair of gapless SSs appears, with one branch merging into conduction bands and the other branch into valence bands, as shown in Fig. 3c. The existence of a single Fermi surface^44^ between 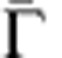 and 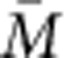 points provides unambiguous evidence of TSSs. Because SSs of the 100% SOC case are adiabatically connected to TSSs of the 350% SOC case, we can conclude that the normal SSs on the Au(111) surface are also TSSs.

### Other noble metals Ag, Cu, Pt and Pd

**Figure 4:**
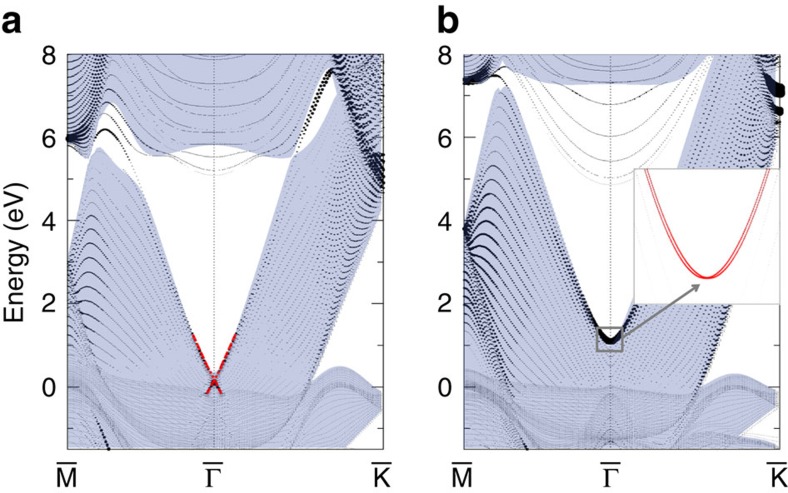
Surface states of Pt and Pd. (**a**) Band structure of Pt (111) surface with normal SOC strength. The size of the filled circles represents the amplitude of projection to the *sp* orbitals of the top two surface atomic layers. Red lines highlight the topological surface states to guide the eyes. (**a**) Band structure of Pd (111) surface with normal SOC strength. The topological surface states are enlarged by filled red circles in the inset. The Fermi energy is shifted to zero and topological surface states remain unoccupied above the Fermi energy.

Ag and Cu exhibit bulk and surface band structures that are very similar to those of Au (see Supplementary Fig. 5) and equivalent in topology. Therefore, we conclude that Au, Ag and Cu are all topological metals wherein TSSs exist on the surface (see Supplementary Fig. 2). We note that the spin splitting observed on Cu(111) is surprising larger^18^ than expected from the Rashba effect based on the weak SOC of Cu (ref. 5), indicating the topological origin of SSs. TSSs should also exist on other facets of these noble metals (including Au) owing to the nontrivial *Z*_2_ index of the bulk. This is consistent with SSs, for example, on (110) and (001) surfaces, reported in the literature (for example, refs 45, 46, and references therein). The topological energy gap above the Fermi energy is similar to the case of a recently discovered oxide TI, BaBiO_3_ (ref. 47), and to another topological metal, Sb. Bulk Sb is a semimetal with a direct energy gap and a nontrivial band structure^27^. As a consequence, TSSs have been observed on special facets such as the Sb (111) and (110) surfaces in experiments^48^,^49^,^50^,^51^. After we clarify the topological feature of Au, Ag and Cu, we further generalize the same idea to other FCC noble metals—Pt and Pd. These two metals show very similar bulk band structure to Au (Supplementary Fig. 6). The main difference is that their Fermi energies are lower than that of Au because Pt and Pd have one fewer valence electron than Au. Therefore, TSSs also exist on both Pt and Pd (111) surfaces, as shown in Fig. 4. Compared with those of the Au surface, these TSSs shift downward towards the bulk bands in energy but still lie above the Fermi energy for both Pt and Pd surfaces. For the Pt (111) surface, the Dirac point of TSSs is found to slightly merge into the bulk bands and forms a surface resonance. In the literature, these empty SSs have been observed and also interpreted as Shockley SSs with Rashba-splitting in photoemission and STS for both Pt^52^,^53^,^54^ and Pd^55^. We note that the positions of TSSs in these reports^52^,^53^,^55^ are consistent with our results. For example, STS revealed the unoccupied SSs of the Pt (111) surface at 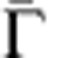 with strong SOC splitting above the Fermi energy^53^. Here we call these SSs on noble metal surfaces topologically derived SSs (TDSSs), to distinguish them from TSSs on a real insulator.

## Discussions

Both TDSSs and Shockley SSs are in-gap states and originate from the inversion of two bulk bands with different symmetries. The TI requires an odd number of band inversions at TRIM in the BZ, and TDSSs are protected by time-reversal symmetry (TRS). In contrast, Shockley states also need band inversions, but they are not limited to finite positions of the zone and to a finite number. Hence, Shockley states may exist more commonly than TDSSs and lack the protection by TRS. The robustness of TSSs, which refers to their existence inside the inverted energy gap in the energy spectra, is protected by the band topology of the bulk. Such topological protection was indeed observed for TDSSs of noble metals in previous experiments. These states remain robust, for example, under the adsorption of alkali metals^56^,^57^,^58^,^59^, guest noble metals^54^, rare gases^60^,^61^, and CO and oxygen^56^,^62^,^63^ and even against surface reconstruction^2^,^64^. In contrast to trivial SSs such as dangling bond states, they usually shift in energy rather than get eliminated by adsorbates or reconstruction^52^. However, the topological protection does not necessarily mean the robustness against any types of electron scattering, for example, by surface defects. Because the Dirac cone is heavily deformed, with both upper and lower cones, as well as bulk bands crossing the Fermi energy, TDSSs can be backscattered on the metal surface, which is different from those of a TI with an energy gap^9^,^10^. This weakness allows versatile manipulation of these SSs by quantum confinement (for example, in quantum corrals). Therefore, we conclude that TDSSs on noble metal surfaces are more stable than trivial SSs in energy spectra due to topological protection. In addition, the Dirac point is expected to open an energy gap by breaking TRS, for example, by depositing magnetic impurities on the surface, as previously observed in magnetic TIs^65^,^66^.

## Methods

### *Ab initio* calculations

The *ab initio* calculations have been performed within the framework of density functional theory with the generalized gradient approximation^67^. We employed the Vienna *ab initio* simulation package with a plane wave basis^68^. The core electrons were represented by the projector-augmented-wave potential. The bulk band structure of Au was projected to Au-*sp* orbitals in Fig. 2c using Wannier functions^69^. The surface band structures were calculated on a slab model that includes thirty-three atomic layers using density functional theory.

### ARPES

The ARPES experiments were performed in an ultrahigh vacuum set-up with a base pressure of 10^−11^ mBar, equipped with a momentum microscope^33^ for the detection of the complete angular distribution of the photoelectrons as a function of their kinetic energy. The Au(111) surface was prepared *in situ* by repeated cycles of Ar^+^ ion sputtering at 2 kV and annealing to 570 K of a mechanically polished (111) crystal. For the 2PPE experiments, we used a commercial OPO laser system (Inspire OPO, Spectra Physics) with a pulse width of 150 fs. The laser was focused on the sample with a spot size of a few micrometres at an incidence angle of 65° with respect to the sample surface. The photon energy was tuned between 4.13 and 4.43 eV, while the light polarization was changed between *s* and *p* using a *λ*/2 plate.

## Additional information

**How to cite this article:** Yan, B. *et al*. Topological states on the gold surface. *Nat. Commun.* 6:10167 doi: 10.1038/ncomms10167 (2015).

## Supplementary Material

Supplementary Information Supplementary Figures 1-6, Supplementary Note 1 and Supplementary References.
