# Supplementary material for: Topological states on the gold surface
Source: Nat Commun. 2015 Dec 14;6:10167. doi: 10.1038/ncomms10167 (PMC4682159; doi:10.1038/ncomms10167)
Supplement: Supplementary Information — Supplementary Figures 1-6, Supplementary Note 1 and Supplementary References. [file ncomms10167-s1.pdf]

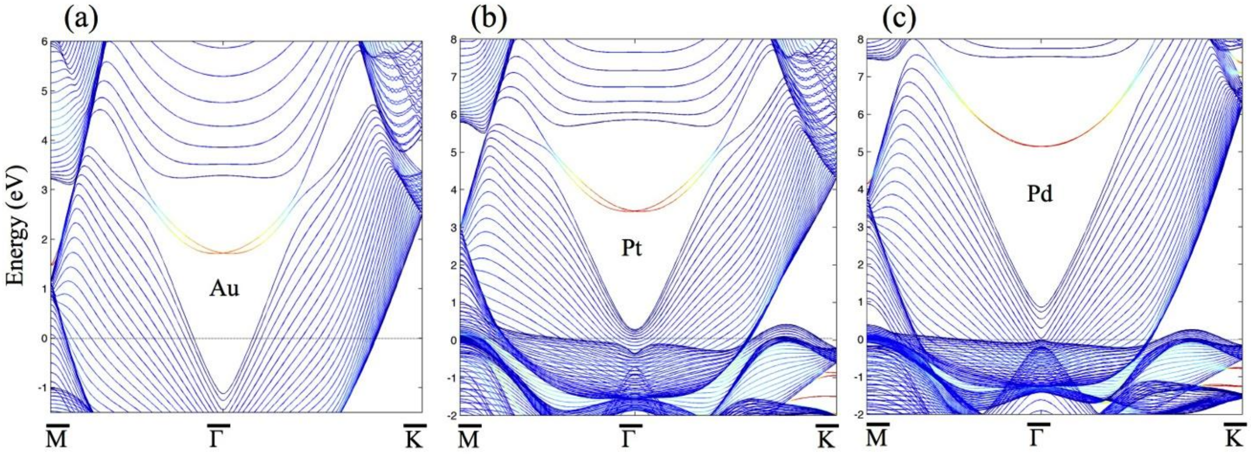

Supplementary Figure 1. Surface band structures calculated from a slab model by tight-binding method. (a) Au, (b) Pt and (c) Pd surface states. The hopping parameters are extracted by projecting corresponding bulk Wannier functions to *spd* states. The color gradient from blue to red represents the increasing contribution of the outer two surface atomic layers. The tight-binding method neglects the effect of surface potential modification due to surface charge redistribution. Therefore, topological surface states (TSSs) exist above the Fermi energy in this case. On a real surface, the surface potential pulls the TSSs down in energy.

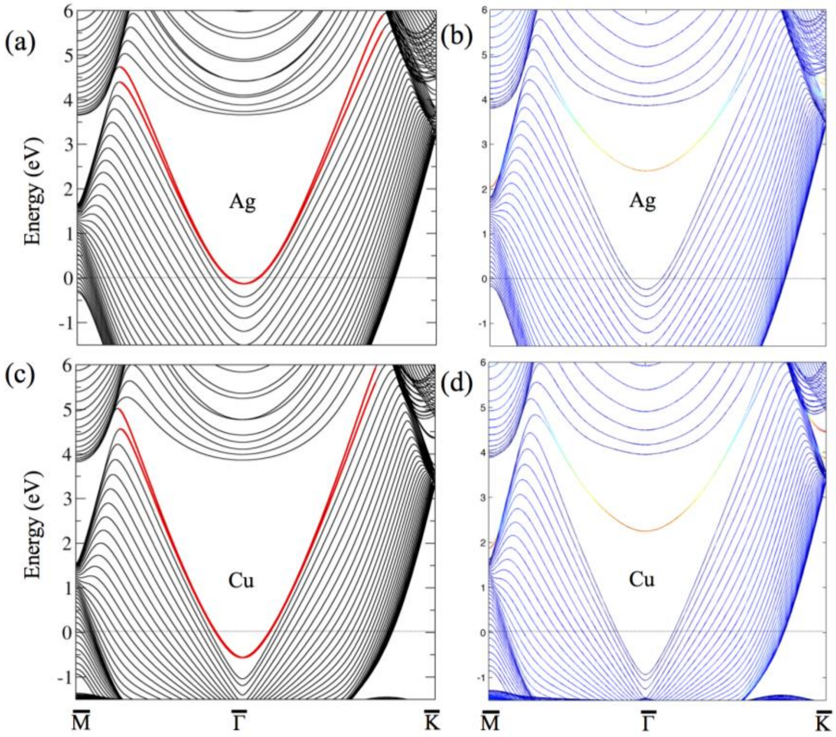

Supplementary Figure 2. Surface band structures calculated from a slab model. (a) *Ab-initio* results for the Ag surface. (b) Tight-binding results based on Wannier functions for the Ag surface. (c) *Ab-initio* results for the Cu surface. (b) Tight-binding results for the Cu surface. The color gradient from blue to red represents the increasing contribution of the outer two surface atomic layers in the tight-binding band structures. TSSs are highlighted by red lines in *ab-initio* band structures. Compared to the tight-binding results, the downshift of TSSs by surface potential is clearly shown in the *ab-initio* band structures.

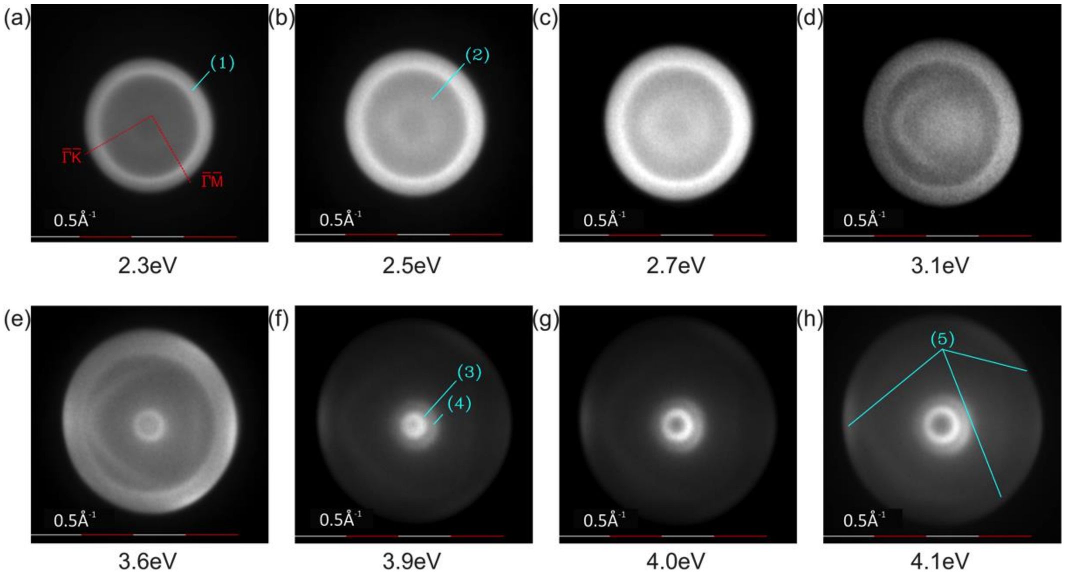

Supplementary Figure 3. Constant intermediate state energy (CISE) maps for the clean Au(111) surface. The data were recorded using *p*-polarized light and a photon energy of 4.27eV.

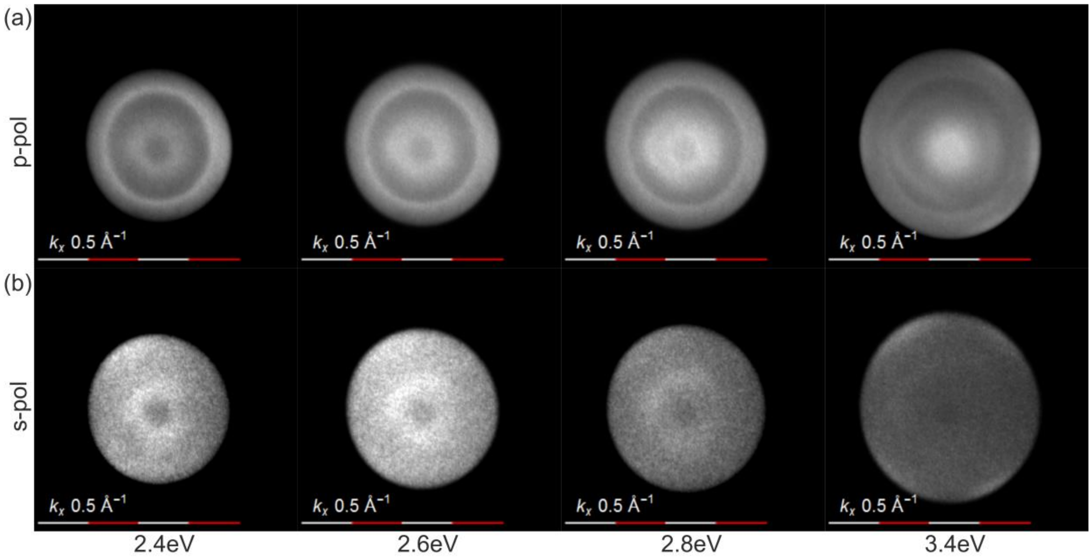

Supplementary Figure 4. Constant intermediate state energy (CISE) maps for the clean Au(111) surface. The data were recorded using *p*-polarized light (a) and *s*-polarized light (b) and a photon energy of 4.27 eV.

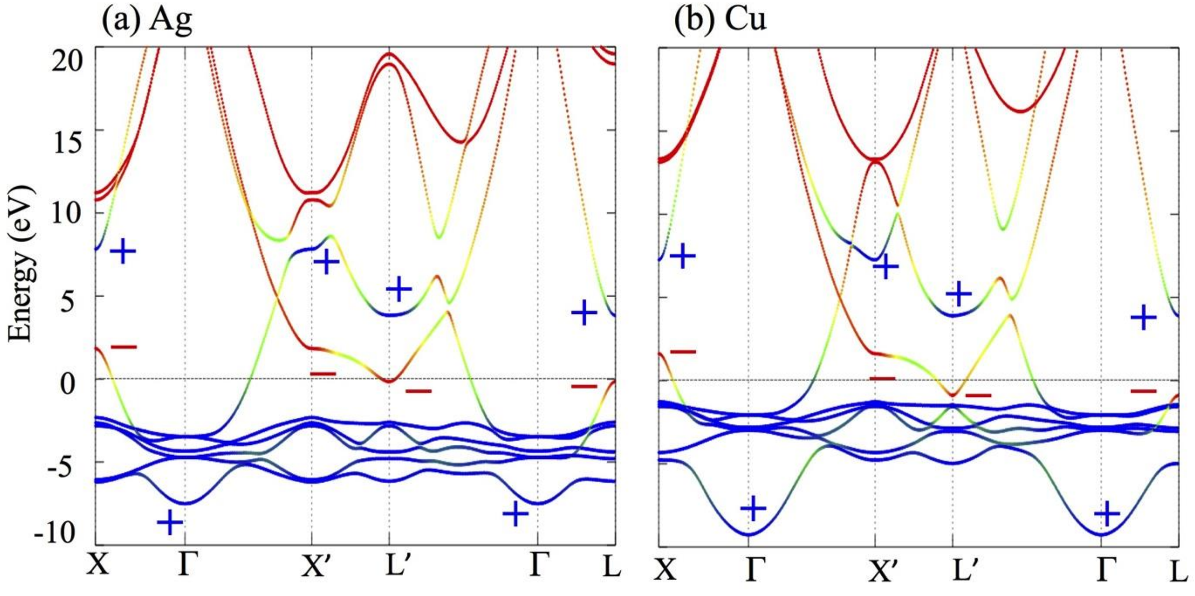

Supplementary Figure 5. Bulk band structures for Ag and Cu. (a) Ag band structure. (b) Cu band structure. The band dispersion is interpolated from Wannier functions that include Ag or Cu-*spd* orbitals. The color gradient from blue to red represents the increasing contribution of Ag- or Cu-*p* states. The Fermi energy is shifted to zero and indicated by the dotted horizontal line. Near the topological energy gap, the *s*–*p* gap above the Fermi synergy, the parity values of correspond bands are labeled by "−" (red, *p* state) and "+" (blue, *s* state). The Z_2 topological invariant calculated from the band structure is $v_0 = 1$ for both Ag and Cu.

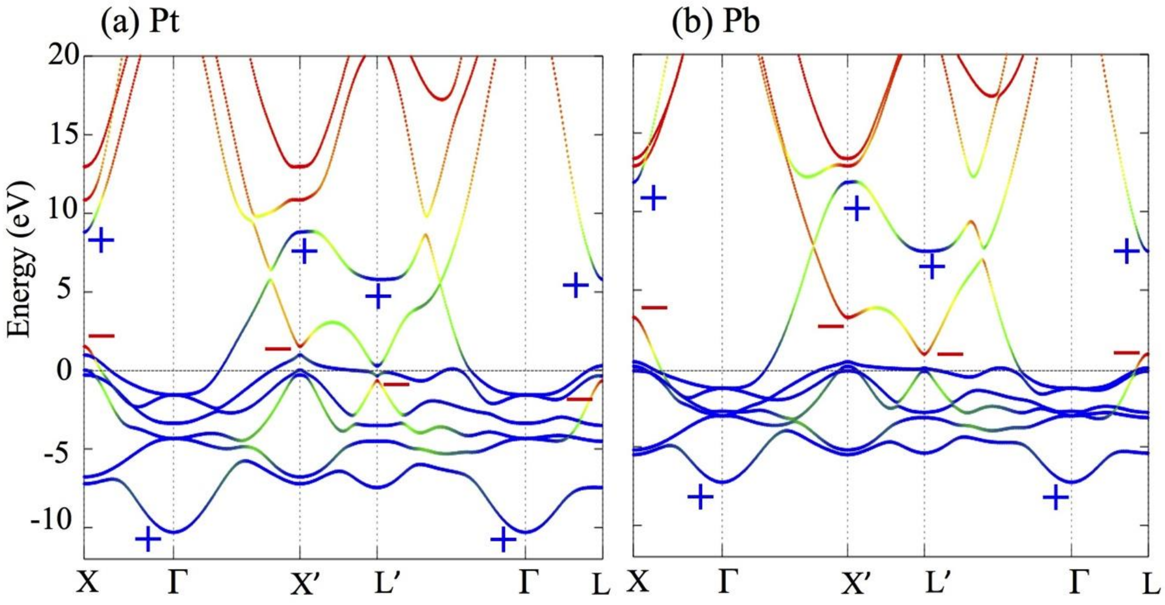

Supplementary Figure 6. Bulk band structures for Pt and Pd. (a) Pt band structure and (b) Pd band structure. The band dispersion is interpolated from Wannier functions that include Pt or Pd-*spd* orbitals. The color gradient from blue to red represents the increasing contribution of Pt- or Pd-*p* states. Besides the Fermi energy is lower than Au, the Z_2 topological invariant calculated from the band structure is $\nu_0 = 1$ for both Pt and Pd with respect to the topological gap above the Fermi energy.

Supplementary Note 1: 2PPE-ARPES experiments

Supplementary Figure 5 shows the angular photoemission distribution for different constant intermediate state energies (CISE) recorded using a momentum microscope 1. The data were recorded with photon energy of 4.27eV and p-polarized light. The high symmetry directions $\bar{\Gamma}\ \bar{K}$ and $\bar{\Gamma}\ \bar{M}$ are marked in the first CISE map as doted and dashed lines, respectively.

For small CISE in Supplementary Figure 5(a), the CISE map reveals a well-defined circular feature (1) with a radius of 0.5Å-1 which is centered around the $\bar{\Gamma}$-point of the surface Brillion zone. As we will argue later, this emission feature can be assigned to the unoccupied part of the TSSs. In addition, the intensity right at the $\bar{\Gamma}$-point is very low which is due to the band gap in the Au bulk band structure in Γ-L direction. This band gap (dark disc at $\bar{\Gamma}$-point) is surrounded by a diffuse circular intensity (2).

Supplementary Figure 5 (b)-(h) show CISE maps from the same data cube for larger CISEs. These data demonstrate the evolution of the ARPES features for increasing CISEs: Feature (1), the unoccupied TSSs, disperses to larger momentum values with increasing the CISE. The diffuse circular intensity (2) surrounding the $\bar{\Gamma}$-point becomes more distinct and finally transforms into a well-defined triangular shaped band structure dispersing to larger k-values with increasing CISE. Such a triangular shaped band structure was already reported for the Cu(111) surface in Ref. 2 and assigned to a bulk transition from an sp-like band into a free-electron like band. Since the bulk band structure for Cu and Au should be rather similar, we also assign the triangular shaped band (2) to a bulk transition.

For intermediate state energies larger than 3.1eV, new bands appear in the ARPES data. The dark disc in the center of the Brillion zone at the $\bar{\Gamma}$-point is replaced by a bright intensity that transforms into two concentric rings (3) and (4) at larger intermediate state energies. We will argue in the following that these two features can be assigned to the occupied part of the TSSs and the unoccupied image potential state, respectively.

In addition, new emission features appear at the outer edge of the photoemission horizon

(5). The emission direction of these features can be correlated to the high symmetry directions of the Au bulk crystal. Therefore, we believe that these emission features (5) can be assigned to bulk signals of the Au band structure or even to the photoemission signal from the Au bulk band edge in ΓL direction.

To confirm our previous assignment of the ARPES features (1), (3), (4) to surface states and of (2), (5) to bulk states, we performed additional ARPES experiments with s-polarized light. A comparison of CISE maps recorded with p-polarized and s-polarized light is shown in Fig. 2 (a) and (b), respectively. While p-polarized light excites electrons from both surface and bulk states, s-polarized light only excites electrons from bulk states. A close look at Fig. 2 immediately confirms our previous assignment. The APRES data recorded with s-polarized light show only the emission features (2) and (5). This result is in line with previous publications for fcc(111) noble metal surfaces [Ref. 2 for (2) and Ref. 3 for (5)].

Furthermore, we performed 2PPE experiments with p-polarized light at different photon energies, varied between 4.13 eV and 4.43 eV. Varying the photon energy allows to disentangle features arising from occupy and unoccupied bands in the ARPES signal. In this way we could determine that feature (3) (inner circle) arises due to the occupied part of the TSS and feature (4) due to the unoccupied image potential state.

The dispersion of the spectral features observed in the 2PPE ARPES measurements is plotted in Fig 2b of the main article. In order to obtain the dispersion, the momentum space position of the occupied and unoccupied TSSs and of the unoccupied image potential state was determined individually by manually fitting a circle to each emission feature in the CISE map. This procedure was repeated for all CISE maps independently. The resulting radii of these circles are plotted as dispersion relation in Fig. 2b of the main article. Clearly, the dispersion relation of the occupied TSS (1) is shifted down in energy by the photon energy to account for its occupied character.

Supplementary References:

1. Krömker, B. *et al.* Development of a momentum microscope for time resolved band structure imaging. *Rev. Sci. Instrum.* **79**, 053702 (2008).

2. Hengsberger, M. *et al.* Photoemission momentum mapping and wave function analysis of surface and bulk states on flat Cu(111) and stepped Cu(443) surfaces: A two-photon photoemission study. *Phys. Rev. B* **77**, 085425 (2008).

3. Woodruff, D. *et al.* Empty surface states, image states, and band edge on Au(111). *Phys. Rev. B* **34**, 764–767 (1986).
